# Ultrasound-Guided Quadrilateral Space Block for the Diagnosis of Quadrilateral Syndrome

**DOI:** 10.1155/2015/378627

**Published:** 2015-01-20

**Authors:** Hamilton Chen, Vincent Reginald Narvaez

**Affiliations:** University of California, Riverside School of Medicine, Riverside, CA 92507, USA

## Abstract

Quadrilateral space syndrome (QSS) is a rare nerve entrapment disorder that occurs when the axillary nerve and posterior circumflex humeral artery (PCHA) become compressed in the quadrilateral space. QSS presents as vague posterolateral shoulder pain that is exacerbated upon the abduction and external rotation of the shoulder. Diagnosis of QSS is difficult because of the vague presentation of QSS. In addition, even though MRI and MR angiography can be used in QSS diagnosis, there is currently no “gold standard” diagnostic imaging studies for QSS. In this case report, we describe a novel ultrasound-guided technique for a diagnostic quadrilateral space block and present a case where the diagnostic block was used to diagnose QSS. We believe that a diagnostic block of the quadrilateral space is a useful adjunct in the evaluation of patients with suspected QSS, especially in cases where examination findings and other diagnostic modalities are indeterminate.

## 1. Introduction

Quadrilateral space syndrome (QSS) is a rare disease caused by the compression of the axillary nerve and possibly the posterior circumflex humeral artery (PCHA) as they course together through the quadrilateral space. QSS is a diagnosis that is difficult to achieve by history and physical exam alone and there are no tests which considered the diagnostic “gold standard” [1].

In some musculoskeletal conditions, due to the low specificity of physical examination maneuvers and lack of a good diagnostic study, local anesthetic blocks often remain as the diagnostic “gold standard” [2, 3]. In these cases, if the administration of a small amount of local anesthetic to the hypothesized pain generator provides significant pain relief for the patient, the diagnosis is established [2].

In this case report, we describe a novel ultrasound-guided technique for a diagnostic quadrilateral space block and present a case where the diagnostic block was used to diagnose QSS. We believe that a diagnostic block of the quadrilateral space is a useful adjunct in the evaluation of patients with suspected QSS, especially in cases where examination findings and other diagnostic modalities are indeterminate.

## 2. Case Presentation

A 42-year-old female with no significant past medical history presented to our outpatient musculoskeletal clinic with left posterior-lateral shoulder pain for 9 months. Prior to her consultation in our clinic, she was evaluated by the orthopedic surgery service, who ordered an MRI of the left shoulder. The MRI revealed edema and enhancement of the teres minor muscle and posterior part of the deltoid muscle. There was also evidence of degeneration of the superior glenoid labrum. The patient desired nonoperative management, so a subacromial bursa steroid injection was performed by the orthopedic surgeon, which was ineffective for pain relief. The patient was then referred to our service for further nonoperative management.

On presentation to our clinic, the patient reported pain in the posterior-lateral shoulder. The pain was described as a constant dull ache without any radiation. Examination of the shoulder revealed a mildly protracted left scapula with no visible atrophy of the shoulder or spine musculature. Range of motion was within normal limits, and results of Neer, Hawkin, Scarf, and Obrien test were negative. There was mild tenderness to palpation at the posterior aspect of the glenohumeral joint inferior to the acromion. Due to the examination findings and MRI evidence of edema in the teres minor and posterior part of the deltoid muscles, we suspected a diagnosis of QSS. An ultrasound-guided local anesthetic block of the quadrilateral space was performed. Subsequent to the block, the patient received 100% relief of her pain and the diagnosis of QSS was confirmed.

## 3. Technique

**Figure 1:**
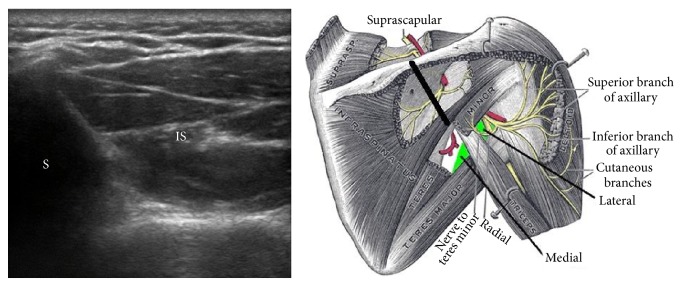
Short axis view of the infraspinatus (IS) at the spine of the scapula (S). Figure adapted from the medical gallery of Mikael Haggstrom.

**Figure 2:**
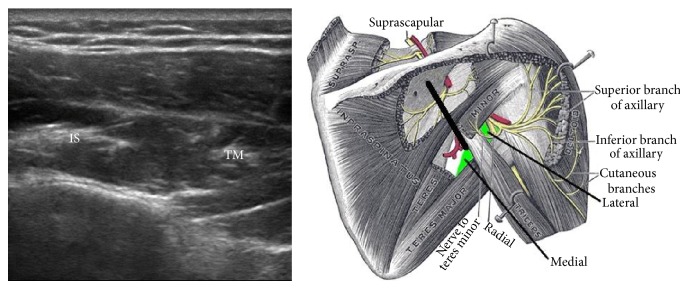
Short axis view of the myotendinous junction of the infraspinatus (IS) and teres minor (TM). Figure adapted from the medical gallery of Mikael Haggstrom.

**Figure 3:**
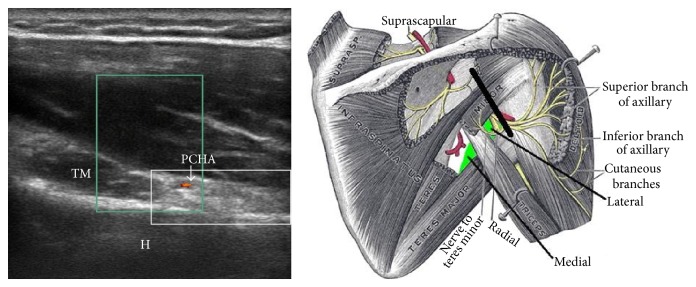
View of the quadrilateral space (white box), posterior circumflex humeral artery (PCHA), humerus (H), and teres minor (TM). Figure adapted from the medical gallery of Mikael Haggstrom.

The patient was placed in a prone position with both upper extremities at her side. The transducer was placed medial to the glenohumeral joint in an orientation perpendicular to the axis of the spine of the scapula (Figure 1). The spine of the scapula was used as a landmark to distinguish between the supraspinatus fossa and the infraspinatus fossa. In the infraspinatus fossa, the infraspinatus and teres minor were visualized in a cross-sectional view at the myotendinous junction (Figure 2). The probe was then moved inferiorly until the teres minor was centered and traced distally until the posterior circumflex humeral artery was visualized. Visualization of the posterior circumflex humeral artery was enhanced by utilizing the Doppler function (Figure 3). In this view, a syringe attached to a 22 gauge 1.5 inch needle containing 3 mL of 1% lidocaine was inserted along the long axis of the ultrasound probe from a cranial to caudal direction. The needle was guided under live ultrasound to the previously identified space to block the axillary nerve. Visualization of the posterior circumflex humeral artery was maintained to avoid inadvertent intravascular injection.

## 4. Discussion

Anatomically, the quadrilateral space is bordered superiorly by the teres minor, laterally by the proximal humerus, medially by the long head of the triceps tendon, and inferiorly by the teres major [4]. These borders create the compartment that the distal branch of the axillary nerve and posterior circumflex humeral artery traverse through. Fibrous bands between the teres major and the long head of the triceps tendon have been frequently implicated as a major cause of the compression in QSS. A prior cadaveric dissection study demonstrated the presence of these “fibrous bands” in 14 of 16 cadavers—the presence of which may predispose individuals to developing QSS [1, 4]. Moreover, there are other causes such as tumors, ganglions, and muscle hypertrophy that can compress the axillary nerve, leading to the signs and symptoms of QSS [4, 5].

The signs and symptoms of QSS are typically secondary to the compression of the axillary nerve. On examination, patients may demonstrate point tenderness in the quadrilateral space [1]. Patients with QSS may also typically report symptoms of poorly localized posterior shoulder pain and paresthesia in the posterior shoulder of the dominant upper extremity. These symptoms will often be aggravated with humeral abduction and external rotation (ABER); thus, patients with QSS can present with shoulder discomfort when performing overhead tasks [6]. The pain associated with QSS can also radiate in a nondermatomal pattern and may also present with some weakness due to teres minor and deltoid muscle atrophy [7].

Diagnosis of QSS using history and physical exam alone is difficult since it may present as shoulder pain that is not necessarily localized to the quadrilateral space. Furthermore, paresthesias may or may not be present in patients suffering from QSS [6]. Additionally, QSS is difficult to diagnose since it may present similarly to other conditions such as thoracic outlet syndrome, rotator cuff pathology, or other shoulder abnormalities [8].

Currently, there is no “gold standard” diagnostic test for QSS. However, magnetic resonance imaging of suspected QSS patients is often used as it may show selective fatty atrophy of the teres minor and possibly the deltoid muscles, both of which are innervated by the axillary nerve [9, 10]. In addition, increased T2 signaling in MRI suggests neurogenic edema which is possibly due to axillary nerve compression. There is no data regarding the sensitivity or specificity of MRIs for QSS. However, a study of 2,436 shoulder referrals showed 19 cases or 0.8% of patients having selective teres minor atrophy together with increased T2 signaling [11].

In addition to MRI, angiography may be used as a diagnostic test for QSS. There is currently no data regarding the sensitivity of angiography in diagnosing QSS. Multiple studies have used subclavian arteriogram or MR angiography to confirm QSS diagnosis [8, 12]. PCHA occlusion is expected in patients with QSS symptoms especially upon the abduction and external rotation of the shoulder. However, a study conducted by Mochizuki et al. showed using MR angiography that 80% of patients asymptomatic of QSS can have occlusion or stenosis of their PCHA when the humerus was abducted [13]. In this regard, angiography may be more useful in diagnosing QSS in patients experiencing clinical symptoms but with no teres minor atrophy evaluated by MRI [9].

With the advancement of ultrasound, sonographic evaluation has also become a recent option to aid with QSS diagnosis. Axillary nerve compression can manifest as teres minor fatty change in ultrasound as well as diffuse increase in teres minor echogenicity, in addition to a small loss of muscle bulk with the absence of rotator cuff abnormalities [6, 14, 15]. More specifically, color Doppler ultrasound can be used to elucidate blood flow in the PCHA and thus quadrilateral space compression, in order to support the diagnosis of QSS [16]. However, there is difficulty in directly examining the axillary nerve since it is small and thus hard to detect in ultrasound.

In the case above, our patient presented with nonspecific signs and symptoms of posterior-lateral shoulder pain. On MRI, the patient had findings of enhancement in the teres minor and posterior part of the deltoid muscles. Even though these findings were suggestive of QSS, the patient had evidence of degeneration of the superior glenoid labrum, a condition which may also manifest as posterior-lateral shoulder pain. These nonspecific findings ultimately led to our decision to perform an ultrasound-guided diagnostic block of the quadrilateral space. After the successful block, we were able to confirm the rare diagnosis of QSS.

We strongly believe that the procedure is an extremely helpful adjunct in the evaluation of patients with suspected QSS. More studies should be conducted to assess the sensitivity and specificity of an ultrasound-guided diagnostic block of the quadrilateral space in the diagnosis of QSS.
